# Rab5 hyperactivation: The central hub for endolysosomal dysfunction in Down syndrome

**DOI:** 10.4103/NRR.NRR-D-25-00967

**Published:** 2025-12-30

**Authors:** Xu-Qiao Chen

**Affiliations:** Department of Neurosciences, University of California San Diego, La Jolla, CA, USA

**Rab5 as a central regulator of the endolysosomal network:** The endolysosomal network (ELN) comprises a dynamic system of membrane-bound organelles responsible for cargo trafficking, degradation, and intracellular signaling, all of which are essential for maintaining cellular homeostasis. Within this system, members of the Rab family of small GTPases serve as key regulators of vesicular transport, marking specific compartments. Among them, Rab5 plays a pivotal role in orchestrating the formation of the early endosome (EE), membrane fusion events, and the transition of EE to downstream stages. Like other Rab proteins, Rab5 cycles between an active GTP-bound state and an inactive GDP-bound state. This cycle is tightly controlled by guanine nucleotide exchange factors, which activate Rab5 by facilitating GDP-GTP exchange, and GTPase-activating proteins, which inactivate it by promoting GTP hydrolysis (Stenmark, 2009; Huotari and Helenius, 2011).

In its active form, Rab5 recruits diverse effectors that regulate endosomal maturation, membrane remodeling, and communication with other downstream organelles, such as the late endosome (LE) and recycling endosome (Stenmark, 2009). Although considered a traffic coordinator for EEs, a pilot study has repositioned Rab5 as a central regulator of organelle biogenesis and ELN integrity by highlighting the indispensable role of Rab5 in organelle formation (Zeigerer et al., 2012), while more recent findings demonstrate that Rab5 hyperactivation can disrupt the endosomal Rab cascade, leading to widespread dysfunction of the ELN, as observed in Down syndrome (DS) (Chen et al., 2025a). These discoveries underscore that Rab5 is not merely a regulator of trafficking but a critical molecular switch, whose dysregulation can lead to systemic collapse of the ELN.

**Figure 1:**
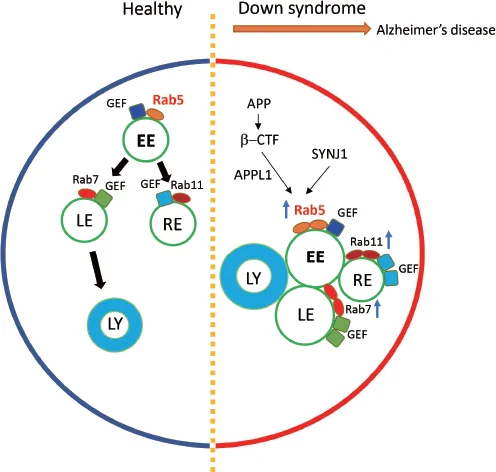
Rab5 functions as a central hub in the endolysosomal network under both physiological and pathological conditions. Under normal conditions (left), Rab5 regulates the progression of early endosome (EE) to late endosome (LE), recycling endosome (RE), and lysosome (LY), acting as a key coordinator of endolysosomal architecture and trafficking. In DS (right), increased APP-mediated accumulation of β-C-terminal fragment (β-CTF) induces Rab5 hyperactivation/EE enlargement in an APPL1-dependent and Alzheimer’s disease-independent manner in neurons, whereas other genes, such as *SYNJ1*, can trigger similar endosomal alterations in a cell type–dependent manner. Rab5 hyperactivation in turn enhances the activity of other endosomal Rabs, including Rab7 (LE) and Rab11 (RE). This results in increased recruitment of Rabs and their guanine nucleotide exchange factors (GEFs) to EEs, as well as elevated levels of lysosomal cathepsins. These changes lock the endosomal system in an immature state, disrupting endolysosomal trafficking and degradation pathways and ultimately contributing to disease progression in Down syndrome.

The physiological significance of Rab5 was compellingly demonstrated by Zeigerer et al. (2012), who combined mathematical modeling with *in vivo* RNA interference in adult mouse livers. This work established that Rab5 is not just one of many contributors to endosomal function—it is indispensable for the biogenesis of the endolysosomal system. Mild reductions of all three Rab5 isoforms (a, b, and c) had minimal consequences, but depletion beyond a threshold triggered a collapse of the entire ELN. This collapse included a marked loss of EEs, LEs, and lysosomes, leading to impaired physiological processes such as low-density lipoprotein uptake and polarized protein sorting in hepatocytes. Importantly, this disruption was not due to reduced expression of downstream effectors, such as endosomal coat proteins. Instead, it reflected a failure to assemble functional endosomal compartments in the absence of sufficient Rab5. These findings support a model in which endosome fusion and fission rates are nonlinear functions of Rab5 activity. Rab5 emerges as a central regulator of endolysosomal architecture and function (**Figure 1**).

**Rab5 hyperactivation disrupts the endosomal Rab cascade in Down syndrome:** While previous studies have shed light on the effects of Rab5 loss-of-function, the consequences of chronic Rab5 hyperactivation, a shared feature in both Alzheimer’s disease (AD) and DS (Nixon, 2017; Chen and Mobley, 2019), have remained less clear. A recent study addressed this question within the context of DS (Chen et al., 2025a), a condition marked by trisomy of human chromosome 21, which includes an extra copy of the amyloid precursor protein (*APP*) gene (Antonarakis et al., 2020). Adults with DS have a markedly increased risk of developing early-onset AD. DS and AD share common cellular features, including endosomal abnormalities such as enlarged EEs. It has been demonstrated that APP-mediated increases in β-C-terminal fragment (β-CTF) lead to early endosome enlargement in an APPL1-dependent manner in neurons (Kim et al., 2016). Other genes, such as *SYNJ1*, can induce similar endosomal alterations in non-neuronal systems such as lymphoblastoid cell lines (Cossec et al., 2012), highlighting the complexity of endosomal pathology in DS. However, the precise mechanisms by which elevated Rab5 activity contributes to endolysosomal dysfunction and AD-related pathology remain unclear.

To investigate this, Chen et al. (2025a) performed an in-depth analysis of postmortem brain tissue from individuals with DS, DS-AD, and cognitively normal controls, as well as from Dp16 mice, a well-established DS model. For the first time, they systematically quantified the activity of multiple endosomal Rab GTPases, revealing widespread Rab5 hyperactivation in both DS-AD and DS brains, which coincided with increased activation of downstream endosomal Rabs, including Rab4, Rab7, and Rab11 (**Figure 1**). Moreover, this dysregulation was strictly dependent on increased *APP* gene dose, as evidenced by its absence in a rare DS case with partial trisomy excluding *APP*, and in gene-corrected Dp16 mice with normalized *App* expression (Chen et al., 2025a).

Mechanistic studies pinpointed the β-CTF product of APP as the key driver of endosomal Rab hyperactivation. Increased β-CTF recruits the adaptor protein APPL1 to EE membranes, thereby facilitating persistent Rab5 activation. This results in excessive accumulation of Rab5 and its downstream Rabs on EEs, stalling the normal progression of the endosomal maturation pathway. The downstream Rab cascade becomes impaired: (1) the transition to LEs and lysosomes is blocked, and (2) trafficking compartments become congested and dysfunctional. This arrest is further characterized by increased expression of selective guanine nucleotide exchange factors, such as those for Rab7 and Rab11, which reinforces a hyperactive yet paralyzed endosomal network.

Additionally, Rab5 hyperactivation led to increased lysosomal fusion events (enlarged lysosomes) and upregulation of lysosomal enzymes such as cathepsins, reflecting compensatory responses that ultimately fail to restore proteostasis (**Figure 1**). The result is a breakdown in cargo degradation, membrane recycling, and receptor signaling—all critical processes for neuronal function.

Importantly, this pathological model contrasts with the regulated Rab5 dynamics observed under physiological conditions, as described by Zeigerer et al. (2012). In healthy cells, Rab5 activation is transient and spatially restricted, ensuring the sorting and degradation of cargoes. In DS and AD, however, persistent Rab5 hyperactivation traps the endosomal system in an immature state, leading to the collapse of endolysosomal trafficking and degradation pathways. Notably, experiments in *App*-knockout neurons demonstrated that Rab5 hyperactivation alone is sufficient to impair endolysosomal function, highlighting its role as a critical pathological hub (Chen et al., 2025a). These findings establish Rab5 as a master regulator whose dysregulation can initiate a cascade of neurodegenerative changes by disrupting endolysosomal homeostasis.

**Limitations and perspectives:** Although previous morphological studies revealed the enlargement of EE and LE, not all endosomal Rabs, when hyperactivated, induce observable changes in compartment morphology—for example, Rab4 and Rab11. Therefore, directly measuring the activity of different Rab proteins represents a more reliable approach. In addition to the biochemical methods used in this study, staining tissue sections with antibodies specific to the GTP-bound forms of various Rabs could provide valuable insights.

Despite its strengths, this study has several limitations. A major limitation is reliance on postmortem brain tissue, which only provides a late-stage snapshot of the disease and cannot resolve the temporal sequence of pathological events. The postmortem interval may influence measurements in postmortem human samples. Although the authors demonstrate that changes in endosomal Rab activity are independent of AD pathology, future studies using longitudinal animal models or patient-derived organoids may help simulate disease progression and clarify the dynamic role of Rab5 in AD.

Moreover, the study focuses exclusively on the frontal cortex of individuals with DS. Given that AD pathology in DS is not regionally restricted but spreads across multiple brain areas, future work should aim to chart the spatial distribution of Rab5 activity across the brain. Mapping these changes in relation to hallmark AD features, such as amyloid plaques and neurofibrillary tangles, will help clarify how endosomal dysfunction evolves and interacts with broader neurodegenerative processes.

Another limitation is that the study does not address when Rab5 hyperactivation begins. A previous histological study has detected early endosomal abnormalities in fetal DS brains (Cataldo et al., 2000), suggesting that Rab5 dysregulation might occur during development. However, it remains unknown whether Rab5 activity changes dynamically with age or how it temporally relates to the onset of amyloid or tau pathology. Longitudinal studies using developmental models or organoids could shed light on these early-stage events.

The use of whole brain tissue also prevents resolution at the cellular level. While this study focuses on neurons, Rab5 is a ubiquitous regulator of membrane trafficking, and its dysfunction in non-neuronal cells, such as microglia, astrocytes, and oligodendrocytes, could also contribute to disease. In particular, altered Rab5 activity in glial cells could influence processes such as cytokine release, synaptic pruning, and myelination, thereby exacerbating neuroinflammation and neuronal vulnerability. Future studies employing single-cell or spatial omics approaches will be critical to define cell-type-specific patterns of Rab5 dysregulation, clarify their impact on neuroinflammatory pathways, and identify potential therapeutic targets. Functionally, Rab5 is known to regulate a broad array of processes beyond EE maturation, including receptor signaling, autophagy, and synaptic vesicle trafficking. These pathways are especially critical in neurons under both physiological and pathological conditions and could mediate diverse aspects of neurodegeneration. In the context of DS, Rab5 hyperactivation could disrupt these processes through altered endosomal–lysosomal dynamics, thereby linking *APP* gene dosage to broader cellular dysfunction. The extent to which each pathway is affected remains to be clarified and represents an important direction for future research.

The authors suggest that targeting APP or Rab5 may rescue ELN dysfunction. Notably, the same research group has recently shown that antisense oligonucleotide-based strategies reducing APP or Rab5 expression can normalize ELN deficits and reverse several AD-related phenotypes in the Dp16 mouse model (Chen et al., 2025b). Although these findings fall outside the scope of the present perspective, they support the mechanistic model proposed here and highlight the translational potential of this research.

Together, these findings support a model in which Rab5, a key coordinator of endosomal trafficking, becomes pathologically hyperactivated in DS due to elevated β-CTF levels. Under physiological conditions, Rab5 acts as a central hub in the ELN, orchestrating cargo sorting, endosome fusion, and maturation in a tightly regulated sequence. However, in disease states, chronic Rab5 activation — similar to that seen with the constitutively active mutant (Rab5^Q79L^) (Chen et al., 2025a) — disrupts the Rab cascade, stalls endosomal maturation, and leads to systemic collapse of the ELN. Thus, a molecule essential for normal intracellular homeostasis becomes a driver of neurodegeneration when dysregulated.

Looking forward, several important research directions emerge. First, there is a need for tools that allow for dynamic and cell-type-specific manipulation of Rab5 activity *in vivo*. Conditional expression of dominant-negative Rab5 or wild-type (Pensalfini et al., 2020) or constitutively active Rab5, or inducible CRISPR-based editing of its regulators, would clarify its causal role in disease mechanisms. Second, therapeutic approaches using antisense oligonucleotides to target *APP* or *Rab5* show promise but require more rigorous preclinical validation. This includes optimization of dose, delivery methods, timing of intervention, and toxicity assessment. Third, identifying the key downstream effectors of Rab5 that mediate its toxic effects will help uncover the molecular mechanisms linking its hyperactivation to multiple aspects of the pathology. Proteomic and genetic screening approaches could be instrumental in this effort. Fourth, cutting-edge imaging techniques, including live-cell microscopy, super-resolution imaging, and biosensor-based assays, in neurons or brain organoids, could reveal the spatial and temporal dynamics of Rab5-driven ELN dysfunction. These tools could help identify early biomarkers or intervention points relevant not only to DS but also to AD.
